# Activity of Some Plant and Fungal Metabolites towards *Aedes albopictus* (Diptera, Culicidae)

**DOI:** 10.3390/toxins13040285

**Published:** 2021-04-18

**Authors:** Sonia Ganassi, Marco Masi, Pasqualina Grazioso, Antonio Evidente, Antonio De Cristofaro

**Affiliations:** 1 Department of Agricultural, Environmental and Food Sciences, University of Molise, Via De Sanctis, 86100 Campobasso, Italy; 2 Department of Chemical Sciences, University of Naples Federico II, Complesso Universitario Monte S. Angelo, Via Cintia 4, 80126 Napoli, Italy; marco.masi@unina.it (M.M.); evidente@unina.it (A.E.); decrist@unimol.it (A.D.C.); 3 Department of Life Sciences, University of Modena and Reggio Emilia, Via Campi 213/D, 41125 Modena, Italy; grazpa04@unimore.it

**Keywords:** Asian tiger mosquito, bioinsecticides, Amaryllidaceae alkaloids, naphthoquinones, benzofuranones, larvicidal activity

## Abstract

*Aedes albopictus* (Skuse) is a widespread mosquito, a vector of important human arboviruses, including Chikungunya, Dengue and Zika. It is an extremely difficult species to control even for the onset of resistances to chemicals insecticides, therefore ecofriendly products are urgently needed. In this study, the activity of Amaryllidaceae alkaloids and some of their semisynthetic derivatives, of 2-methoxy-1,4-naphthoquinone and two analogues, of cyclopaldic acid and *epi*-epoformin on the survival and development of *Ae. albopictus* larvae was evaluated. First-instar larval exposure for 24 and 48 h to cyclopaldic acid, resulted in mortality mean per-centage of 82.4 and 96.9 respectively; 1,2-*O*,*O*-diacetyllycorine 48h post-treatment caused 84.7% mortality. Larval and pupal duration were proved to decrease significantly when larvae were exposed to cyclopaldic acid, 1,2-*O*,*O*-diacetyllycorine and *N*-methyllycorine iodide. The mean number of third-instar larvae surviving to 2-methyl-1,4-naphthoquinone, 2-hydroxy-1,4-naphthoquinone and 2-methoxy-1,4-naphthoquinone was significantly lower than the number of correspondent control larvae over the time. This study indicated that 1,2-*O*,*O*’-diacetyllycorine, *N*-methyllycorine iodide, cyclopaldic acid and 1,4-naphthoquinone structural derivatives have good potential for developing bioinsecticides for mosquito control programs. The obtained results are of general interest due to the global importance of the seri-ous human diseases such a vector is able to spread.

## 1. Introduction

*Aedes albopictus* (Skuse) (Diptera: Culicidae), commonly known as the “Asian tiger mosquito”, is an invasive species which spread globally from its native range in Asia to both tropical and temperate regions [1,2]. It is a vector of several important arboviruses, including Chikungunya, Dengue and Zika virus (ZIKV), and its role in different outbreaks worldwide has been reported, making it a major threat to public health [3,4,5,6,7]. The transmission of this disease is increased even if great efforts are made to control arbovirus infections, such as Dengue [6]. Furthermore, the ability of *Ae. albopictus* to carry and transmit ZIKV, has been established [7,8,9]. Mosquito control is the only way to reduce the transmission risk to protect human populations from mosquito-transmitted diseases without an efficient vaccine [5,10,11,12]. Nevertheless, this seems an extremely difficult task, as *Ae. albopictus* exploits a variety of water-collecting containers.

The control of *Ae. albopictus* mainly relies on the reduction of larval breeding sites, insecticides or biological agents [13]. Non-conventional methods, such as the use of irradiated or genetically modified mosquitoes and *Wolbachia* infection, are under implementation and may be used in the future [14,15,16]. Insecticides can be used against both adult mosquitoes and larvae in forms of space treatment, indoor residual spraying, and as larvicides [5,17,18]. However, insecticide resistance, already widespread in *Aedes aegypti* (L.), is increasing in *Ae. albopictus*. Moreover, considering the very limited number of insecticides available on the market for public health, many concerns are raised regarding the global fight against the transmission of diseases [19,20]. In recent years, a renewed interest in biologically active compounds from natural sources has emerged, due to the variety of their biological activities and potential practical applications in different fields [21,22,23,24,25,26,27]. Furthermore, the use of natural ecofriendly biopesticides is a strong and urgent request coming from consumers and from the authorities [22,27]. Natural products, of plant and fungal origin, may offer a wide source of active compounds from which to select environmentally friendly alternatives as mosquito control agents. Amaryllidaceae alkaloids and their synthetic derivatives are well known to have a wide range of biological and pharmacological activities [28,29]. Lycorine, the main Amaryllideaceae alkaloid isolated from *Sternbergia lutea* (L.) Ker-Gawler [30], which is its best source (11g/dried plant kg), exhibits antitumor and antiviral activity [31]. Furthermore, it acts as a powerful inhibitor of ascorbic acid biosynthesis and of cell growth and cell division, including antitumor activity in animal and human cell lines [32]. The derivative *N*-methyllycorine iodide completely suppresses HeLa cell invasion of type I collagen, in vitro at nontoxic concentrations [28]. *N*-methyllycorine iodide, 1,2-*O*,*O*’diacetyllycorine, α-dihydrolycorine, lycorine hydrochloride, and lycorine-2-one, all derivatives from lycorine and ungeremine, isolated from *Pancratium maritimum* L. [33], but also synthesized by oxidation from lycorine [34], were evaluated together with lycorine for algicidal, bactericidal, fungicidal, herbicidal and insecticidal activities [22].

Despite numerous studies concerning the phytochemistry and pharmaceutical activities, and applications of Amaryllidaceae alkaloids [35,36], their potential for the control of medically important insects has been poorly investigated. Only recently, some Amaryllidaceae alkaloids, such as crinsarnine and sarniensinol and sarniensine, three new crinine and mesembrine type alkaloids, isolated from the South African plant *Nerine sarniensis* Herbert, have shown activity against the ZIKV vector *Ae. aegypti* [37,38].

Among fungal metabolites, *epi*-epoformin, a cyclohexene epoxide isolated from the culture filtrates of *Diplodia quercivora* Linaldeddu and A.J.L. Phillips, a pathogen for cork oak in Sardinia, Italy, showed multiple biological activities, including antifungal, zootoxic and phytotoxic activity [39,40,41]. Cyclopaldic acid, a benzofuranone produced by several fungi belonging to different genera [42,43], shows a wide and different range of biological activities, such as antifungal activity [44,45], inhibits electron transport and oxidative phosphorylation in plant mitochondria [44,46] and also inhibits esterase activity in vitro [47].

Among the quinones, plant derived substances, 1,4-naphthoquinone structural derivatives, showed larvicidal activity against *Ae. aegypti*, *Culex pipiens pallens* Coquillet and *Ochlerotatus togoi* (Theobald) fourth-instar larvae [48]. In particular, among 23 compounds, belonging to different classes of natural compounds, 2-methoxy-1,4-naphthoquinone, isolated together with glanduliferins A and B, two new glucosylated steroids and α-spinasterol from *Impatiens glandulifera* Royle, a plant native to Himalaya [49], showed larvicidal activity against *Ae. aegypti* larvae [50] and anticancer activity in vitro in the single digit micromolar range on three cell lines [49].

In order to find new larvicidal biopesticides, some plant and fungal metabolites, belonging to different chemical classes of natural compounds, such as Amaryllidaceae alkaloids, naphthoquinones, some of their derivatives and the fungal phytotoxins cyclopaldic acid and *epi*-epoformin, were evaluated against *Ae*. *albopictus* larvae. Moreover, the authors investigated whether the alkaloids derivatives, with larvicidal activity, could also affect *Ae*. *albopictus* development.

## 2. Results

**Figure 1:**
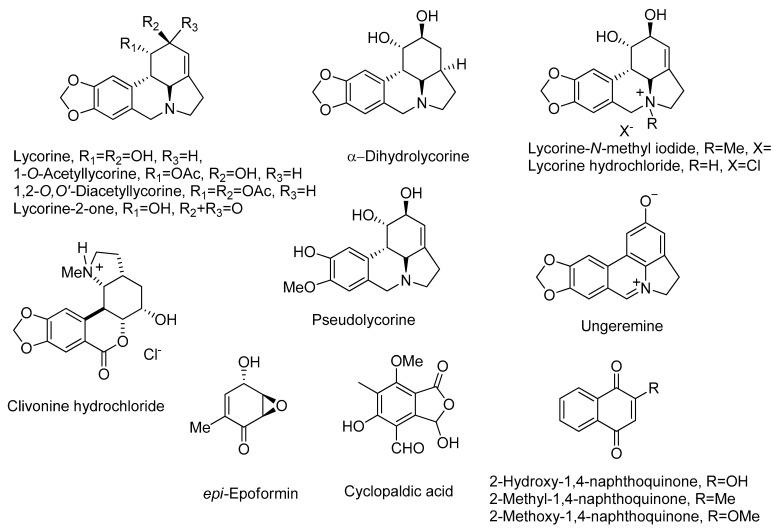
The structure of natural compounds, of the lycorine semisynthetic derivatives, and of the commercially available analogues of 2-methoxy-1,4-naphthoquinone.

The natural compounds, the lycorine semisynthetic derivatives and the commercially available analogue of 2-methoxy-1,4-naphthoquinone used in this study are reported in Figure 1.

The results of the initial exposure screening for 48 h of *Ae. albopictus* first-instar larvae to *epi*-epoformin, clivonine hydrochloride, 1-*O*-acetyllycorine, lycorine-2-one, pseudolycorine, ungeremine, lycorine chlorohydrate, cyclopaldic acid, 1,2-*O*,*O*-diacetyllycorine, *N*-methyllycorine iodide, α-dihydrolycorine at the concentration of 100 ppm, indicated that only four compounds had a larvicidal activity: cyclopaldic acid (96.7%), 1,2-*O*,*O*-diacetyllycorine (80.0%), *N*-methyllycorine iodide (68.0%) and α-dihydrolycorine (40.0%). No mortality was detected after exposure to distilled water and DMSO 1%.

**Figure 2:**
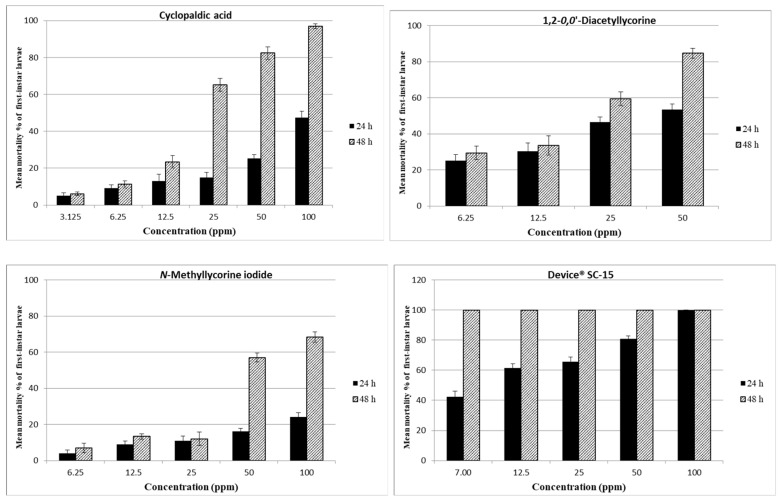
Larvicidal activity of cyclopaldic acid, 1,2-*O*,*O*’-diacetyllycorine, *N*-methyllycorine iodide, Device^®^SC-15 against *Ae*. *albopictus* first-instar larvae. The larval mortality percentages, obtained at 24 and 48 h, were reported as an average of values, from five replicates, corrected using Abbotts’s formula.

The 24 and 48 h larvicidal activity of cyclopaldic acid, 1,2-*O*,*O*-diacetyllycorine, *N*-methyllycorine iodide, α-dihydrolycorine and Device^®^ SC-15, tested at increasing dosages against the first-instar larvae, are presented in Figure 2, Table 1 and Table S1. A strong larvicidal activity was exhibited at 48 h post-treatment by cyclopaldic acid at 50 and 100 ppm, (82.4 and 96.9%) and at 48 h LC_50_ and LC_90_ was 40.1 and 105.2 ppm, LC_50_ and LC_90_ was 28.1 and 60.3 ppm without correction for mortality and by 1,2-*O*,*O*-diacetyllycorine at 50 ppm (84.7%). Device^®^ SC-15 caused a strong larvicidal activity at 50 and 100 ppm (80.8 and 100.0%) at 24h, the mean mortality was 100.0% for all the concentrations tested at 48 h. At 24 h post-treatment, Device^®^ SC-15 LC_50_ and LC_90_ were 18.9 and 104.8 ppm, respectively, and LC_50_ and LC_90_ were 8.8 and 59.3 ppm without correction for mortality.

The effects of cyclopaldic acid, 1,2-*O*,*O*’-diacetyllycorine, *N*-methyllycorine iodide and α-dihydrolycorine on *Ae*. *albopictus* development were presented in Table 2. Observations on the entire larval and pupal duration showed that, at the highest concentrations, they produced over 98% larval mortality except for *N*-methyllycorine iodide 50 ppm that was 75%. The latter compound, at 50 ppm, caused 48% of pupal mean mortality (Table 2).

The Student’s *t*-test carried out on larval duration values, obtained in the control bioassays with distilled water and DMSO 1%, revealed that the difference between the two variables were not statistically significant (*p* > 0.05). The same result (*p* > 0.05) was obtained comparing pupal duration.

Kruskal-Wallis test followed by pairwise Mann-Whitney U-test comparisons revealed significant differences in the larval duration values obtained in bioassays with DMSO 1% and with each of the three compounds tested at different concentrations (DMSO 1%-cyclopaldic acid *H* = 16.4; df 4; *P* = 0.003; DMSO 1%-1,2-*O*,*O*-diacetyllycorine *H* = 9.0; df 3; *P* = 0.029; DMSO 1%-*N*-methyllycorine iodide *H* = 19.5; df 4; *P* = 0.001). The same test showed no significant differences in the larval duration values obtained with DMSO 1% and α-dihydrolycorine (*H* = 4.4; df 3; *P* = 0.22) (Table 2). The same statistical test revealed significant differences in the pupal duration values obtained in bioassays with DMSO 1% and with each of the three compounds tested at different concentrations (DMSO 1%-cyclopaldic acid *H* = 31.9; df 4; *P* = 0.00; DMSO 1%-1,2-*O*,*O*’-diacetyllycorine *H* = 18.1; df 3; *P* = 0.00; DMSO 1%-*N*-methyllycorine iodide *H* = 15.4; df 4; *P* = 0.004). No significant differences were detected in the pupal duration values obtained with DMSO 1% and α-dihydrolycorine (*H* = 2.9; df 3; *P* = 0.399) (Table 2).

**Figure 3:**
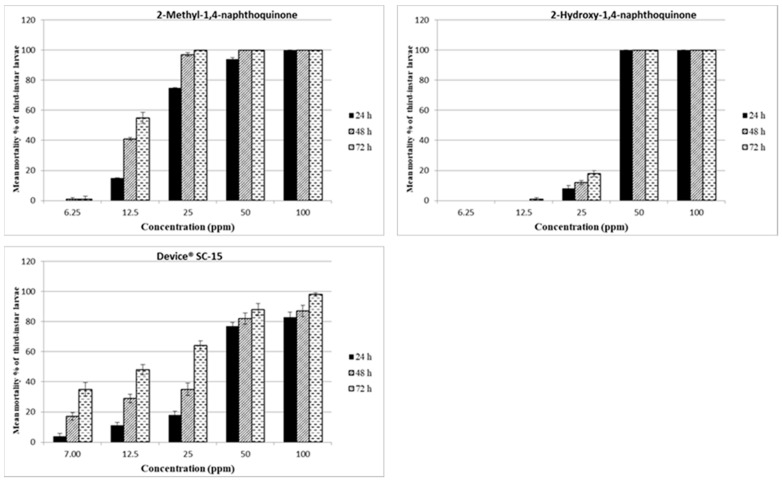
Larvicidal activity of 2-methyl-1,4-naphthoquinone, 2-hydroxy-1,4-naphthoquinone and Device^®^ SC-15 against *Ae*. *albopictus* third-instar larvae. The larval mortality percentages, obtained at 24, 48 and 72 h, were reported as an average of values, from five replicates.

The dose-response mean mortality percentages, the LC_50_ and LC_90_ obtained in naphthoquinones and Device^®^ SC-15 bioassays towards three-instar larvae, are provided in Figure 3, Table 3 and Table S2.

2-Methyl-1,4-naphthoquinone, 2-hydroxy-1,4-naphthoquinone, at 50 and 100 ppm, led to >94% mortality already 24 h after the start of the bioassays. Moreover, 2-methyl-1,4-naphthoquinone at 25 ppm caused >95% mortality after 48 h. 2-Methoxy-1,4-naphthoquinone led to 100% mortality already at 24 h, but only at 100 ppm (Table S2). Device^®^ SC-15 caused >80% mortality at 100 ppm 24 h after the start of the bioassay, and at 50 and 100 ppm at 48h. 2-Methyl-1,4-naphthoquinone exhibited larvicidal activity with 24, 48 and 72 h LC_50_ values of 24.2, 14.5, 12.1 ppm, and LC_90_ of 39.4, 20.7, 15.8 respectively. The LC_90_ values of 2-hydroxy-1,4-naphthoquinone relating to the same time intervals, were 41.0, 39.2, and 39.6 ppm. Device^®^ SC-15 LC_90_ values were 95.3, 90.6, 58.9 ppm (Table 3).

The raw data obtained by larvicidal bioassays carried out on third-instar larvae with 1,4-naphthoquinone structural derivatives and Device^®^ SC-15, tested at the concentrations: 7, 12.5, 25, 50 and 100 ppm, were analyzed using the GLM repeated measures procedure and Bonferroni test. GLM assessed whether the interaction between both test conditions (treatment and control) and the changes over the time of the number of larvae survived to exposure to compounds tested, or the number of survived control larvae, was statistically significant.

The analysis of the data obtained with 2-methyl-1,4-naphthoquinone at concentrations 50 ppm; 2-hydroxy-1,4-naphthoquinone at 25 ppm, 2-methoxy-1,4-naphthoquinone at 50 ppm and Device^®^ SC-15 at all concentrations tested, revealed time × treatment interaction effect (*p* < 0.01) (Table 4).

Indeed, the number of larvae surviving to compounds and product exposure significantly decreases over the time of the bioassay. The analysis of the data obtained with compounds and product tested at all the other concentrations, revealed no time × treatment interaction effect (*p* > 0.05) (Table 4), showing that the number of larvae surviving to compounds exposure does not significantly decrease over the time of the bioassay. In particular, for 2-methyl-1,4-naphthoquinone, at 12.5, 25, 50, 100 ppm, for 2-hydroxy-1,4-naphthoquinone, at 25, 50 and 100 ppm, and for 2-methoxy-1,4-naphthoquinone at 50 and 100 ppm, the differences between the mean number of larvae survived to exposure of the same compound were statistically significant compared to the control from the beginning to the end of the bioassay. For the latter compound, at 25 ppm, the difference was significant only starting from 72 h. For what concerned Device^®^ SC-15, at 7 ppm, the differences between the mean number of larvae survived to exposure to compound was statistically significant compared to the control, only starting from the 48 h. At 12.5, 25, 50 and 100 ppm the differences between the mean number of larvae survived to exposure to the product were statistically significant compared to the control from the beginning to the end of the bioassay. The Bonferroni test was used to assess whether the mean number of larvae that survived the exposure to compounds and Device^®^ SC-15 was significantly smaller than the mean number of the surviving larvae in the control solution, over time, indicating a larvicidal effect of the compounds and of the product. This test revealed that the mean number of surviving larvae exposed to: 2-methyl-1,4-naphthoquinone at 12.5, 25, 50, 100 ppm, 2-hydroxy-1,4-naphthoquinone at 25, 50, 100 ppm, 2-methoxy-1,4-naphthoquinone at 50, 100 ppm and to Device^®^ SC-15 at all concentrations tested, was significantly smaller than the number of correspondent control larvae, respectively, over time (Table 4).

## 3. Discussion

In this study, alternatives to synthetic insecticides towards *Ae. albopictus* larvae have been explored testing natural compounds of different origins and belonging to different chemical classes. Among the alkaloids semisynthetic derivatives tested, only 1,2-*O*,*O*’-diacetyllycorine, *N*-methyllycorine iodide and α-dihydrolycorine showed, for the first time, mosquito larvicidal activity. They also exhibited different degrees of effectiveness, the first two compounds proving to be the most active (84.7 and 68.3% mean mortality) toward *Ae. albopictus* first-instar larvae after 48h of treatment. Amaryllidaceae alkaloids and their derivatives have been reported to exhibit a wide spectrum of bioactivities such as antiproliferative activity, perhaps due to the disruption of eukaryotic protein biosynthesis [51,52,53], and apoptosis inducers [28,54], antitumor, antiviral, acetylcholinesterase inhibitory and cytotoxic activities [29]. Furthermore, very recently, 2-*O*-acetyllycorine was proved to have a marked antiprotozoal activity against *Trypanosoma brucei brucei* (Plimmer and Bradford) [55]. Such a broad spectrum of activity could explain the good larvicidal activity demonstrated by 1,2-*O*,*O*’-diacetyllycorine and *N*-methyllycorine iodide toward *Ae. albopictus* larvae. More recently*,* Han et al. [56] showed that Amaryllidaceae alkaloids, including lycorine, exhibited considerable aphicidal activity and *N*-allylnorgalanthamine displayed a significant inhibition of AChE in *Aphis citricola* van der Goot both in vivo and *in vitro*. Among the compounds tested that were found not to be active towards *Ae. albopictus* larvae, ungeremine was also proved to not be active towards *Ae. aegypti* first-instar larvae [38].

The fungal metabolite cyclopaldic acid showed its effectiveness on larvae, not only at the two major concentrations 50 and 100 ppm (82.4 and 96.9% mean mortality), but also at 25 ppm at which larval mortality was 65.1% after 48h from the start of the bioassay. Furthermore, at the higher concentrations tested (50, 100 ppm), the larval mortality values were comparable with these obtained with the Device^®^ SC-15. Cyclopaldic acid, as well as some other fungal metabolites belonging to different classes of natural compounds, such as seiridin, sphaeropsidin A and payracillic acid, showed both larvicidal and biting deterrent activity against *Ae. aegypti*, a primary vector of Dengue, Yellow Fever and ZIKV [57,58]. Following these results, cyclopaldic acid could provide different management opportunities in different mosquito species control. Some other, mainly natural, phenols were evaluated as potential attractants of *Ceratitis capitata* (Wiedemann) male, the Mediterranean fruit fly [59]. Recently, α-costic acid, a well-known sesquiterpenoid isolated from the native Mediterranean plant *Dittrichia viscosa* (L.) Greuter, had showed a significant acaricidal activity against *Varroa destructor* Anderson and Trueman, the parasite mite of *Apis mellifera* L., the Western or European honeybee [60]. Moreover, costic acid isomers contained in *n*-hexane extracts of the same plant have been held accountable for the contact toxicity against granary weevil adults *Sitophilus granarius* (L.) [61].

To our knowledge, there are no studies describing the effects of the tested Amaryllidaceae alkaloids derivatives on *Ae. albopictus* larval and pupal development. In this regard, cyclopaldic acid, 1,2-*O*,*O*’diacetyllycorine and *N*-methyllycorine iodide, tested on first-instar larvae, caused a significant increase of the larval stage duration at almost all the concentrations tested, while the effect on the pupal stage duration seems to be less marked. However, cyclopaldic acid and *N*-methyllycorine iodide affected pupal viability, causing 38.1% and 48.0% of mean mortality, respectively. The effects on larval and pupal development of these compounds may be due to their growth regulating effects on larvae, which resulted in increasing the larval stage duration and pupal mortality.

The role of alkaloids in insect growth and development was explored by some other authors. Alkaloids extracted from *Annona squamosa* L. (Annonaceae) provoked the death of larvae, pupae and adults of *Anopheles stephensi* Liston, an important vector of malaria. The total developmental period was slightly reduced compared to the control; furthermore, exposed larvae eclosed adult females with reduced fecundity and fertility [62]. In reports by Sun et al., [63] the treatment of *Spodoptera litura* (Fabricius) with *Cynanchum mongolicum* (Maximowicz) (Asclepiadaceae) extracts led to more than half of the resulting pupae not moulting into adults, and also the developmental time, particularly from the third instar to emergence, was increased. Ge et al. [64] also proved that alkaloids from the same plant species had effects on the growth and development of *S. litura*; in fact, higher alkaloid concentrations caused greater developmental disruption and mortality, mainly by 72 h post-treatment. Furthermore, the ecdysone titre of treated larvae and pre-pupae decreased with increasing alkaloid concentration and hormone balance disruption was very similar to that caused by azadirachtin.

The 1,4-naphthoquinones tested proved their larvicidal activity against third-instar larvae. Among the investigated 1,4-naphthoquinone structural analogues, 2-methyl-1,4-naphthoquinone showed remarkable larvicidal activity at concentrations of 25, 50 and 100 ppm (75%, 94% and 100% mean mortality, respectively), after only 24 h of exposure. 2-Hydroxy-1,4-naphthoquinone also exhibited good larvicidal activity with LD_50_ and LD_90_ of 33.7 and 41.0 ppm, respectively, at 24 h of exposure, while 2-methoxy-1,4-naphthoquinone is effective at the highest concentration at 24 and 48 h. The latter compound caused a comparable larvicidal activity value when tested on *Ae. aegypti* first-instar larvae [64] and its activity was also proved against *Ae. aegypti* fourth-instar larvae [48].

Naphthoquinones, in addition to demonstrating good insecticide activity against different species of mosquitoes, are also active against other species of insects and mites [65,66,67].

Our bioassays also indicated that larvicidal activity depends not only on concentration and exposure time, but also on functional groups linked to naphthoquinones. Indeed, at the same concentration, the naphthoquinones with different functional groups have shown a different effectiveness on larval viability. The importance of the functional group in carrying out the activity has also been highlighted in other papers concerning the toxic activity of naphthoquinones against fourth instar *Ae*. *aegypti*, and freshwater snail *Biomphalaria glabrata* (Say) [48]. The authors not only proved the larvicidal activity of 2-methyl-1,4-naphthoquinone but also showed the relationship between the bromonaphthoquinones activity and bromine and other substituents. Kim and Lee [68] also determined the structural toxicity relationships of 5-hydroxy-2-methyl-1,4-naphthoquinone and its structural derivatives, against *Ae. aegypti*, *Cx. pipiens pallens*, and *Oc. togoi* larvae.

Our study also showed that the larvicidal activity of cyclopaldic acid, of some of the alkaloid derivatives and of 1,4-naphthoquinone structural analogues tested, was similar to that of Device^®^ SC-15.

The promising results obtained make these natural compounds worthy of consideration as a bioinsecticide to control *Ae. albopictus* larvae, particularly mosquito larval populations resistant to synthetic chemical insecticides. However, further studies are required to verify their activity and possible toxic effects on non-target organisms when they are applied to natural habitats, since these properties have not yet been investigated. For this purpose, a suitable and effective bioformulation should also be realized. Such compounds could be utilized in larval breeding sites, including rain-water collection areas, peridomestic water, containers and so forth, both in urban places and rural areas. Further investigations are needed not only to determine their potential risks to non-target organisms, but also to the environment in general, including proof of their safety for humans. Nevertheless, our results can be useful for designing vector control strategies against *Ae. albopictus* to avoid spreading significant human diseases.

## 4. Conclusions

1,2-*O*,*O*’-diacetyllycorine, *N*-methyllycorine iodide, α-dihydrolycorine, cyclopaldic acid and 1,4-naphthoquinone structural derivatives demonstrate strong larvicidal activity against *Ae. albopictus* larvae. Besides causing larval mortality, cyclopaldic acid, 1,2-*O*,*O*’-diacetyllycorine and *N*-methyllycorine iodide induce a significant increase of the larval stage duration. The production of all these compounds should easily scale up at the industrial level. In fact, lycorine derivatives can be semisynthesized from lycorine, which can be obtained as a crystalline compound and in high yield by an ecofriendly process from wild *Sternergia lutea* Ker Gawl, a plant which could also be extensively cultivated, as well as *Impatiens glandulifera* Royle, to obtain 2-methoxy-1,4-naphthoquinone. Cyclopaldic acid similarly could be obtained as a white crystal in high yield by the fermentation of *S. cupressi* and its successive crystallization from water. Thus, after investigation of the ecotoxicology effects of the most active compounds, their efficacious bioformulation could be developed to obtain bioinsecticides with potential practical applications for the control of mosquitoes.

## 5. Materials and Methods

### 5.1. Insects

First and third-instar larvae of *Ae. albopictus*, reared in 2 L plastic jars containing distilled water (1L) and 50 g of insect diet (50% of tuna fish flour, 50% of bovine liver powder and a standard dose of Vitamin Mix equal to 0.4 g in 100 mL of solution) were purchased from Centro Agricoltura Ambiente “G. Nicoli” (Crevalcore, Bologna, Italy), where the mosquito strain used for the study was reared for 63 generations under controlled conditions [69]. Jars with larvae were maintained at 27 ± 2 °C, 90 ± 5% relative humidity (R.H.), 14:10 L:D photoperiod.

### 5.2. Natural Compounds Tested

Lycorine was obtained from acid extraction of *S. lutea* bulbs collected in Apulia coast [30]; 1,2-*O,O’*-diacetyllycorine, 1-*O*-acetyllycorine, lycorine-2-one and α-dihydrolycorine were prepared from lycorine as previously reported [70] as well as lycorine chlorohydrate [40]. Clivonine hydrochloride was kindly supplied from Professor C. Fuganti, Istituto di Chimica, Politecnico di Milano, Italy as well as *N*-methyl lycorine iodide was a generous gift from Professor H. M. Fales, Department of Health, Education and Welfare, Bethesda, MD 20014, U.S.A. Ungeremine was extracted from bulbs of Egyptian *P. maritimum* [33] and also obtained by Se_2_O oxidation from lycorine [34]. Pseudolycorine was obtained from bulbs of *Narcissus tazetta* subsp. *tazetta* L., collected in Turkey [71]. *Epi*-epoformin was extracted from the culture filtrates of *D. quercivora* [39]. 2-methoxy-1,4-naphthoquinone was obtained from the organic extract of *I. glandulifera* [49]. 2-Methyl-1,4-naphthoquinone and 2-hydroxy-1,4-naphthoquinone were purchased from Sigma-Aldrich, Milan, Italy. Cyclopaldic acid was extracted from the culture filtrates of *S. cupressi* [44]. The purity (>98%) of the natural and semisynthetic compounds was ascertained by HPLC, ^1^H NMR and ESI MS spectra.

### 5.3. Larvicidal Tests

The larvicidal activity of the compounds was evaluated according to WHO standardized procedures and guidelines for larvicidal testing [72]. An initial screening with *ep*i-epoformin, clivonine hydrochloride, 1-*O*-acetyllicorine, lycorine-2-one, pseudolycorine, ungeremine, lycorine chlorohydrate, cyclopaldic acid, 1,2-*O*,*O*’-diacetyllycorine, *N*-methyllycorine iodide and α-dihydrolycorine, tested at concentration of 100 ppm, was carried out. Twenty-four replicates, each consisting of 5 first-instar larvae, were utilized for each compound concentration as well as for the controls. Since dimethyl sulfoxide (DMSO) 1% was used to solubilize the compounds tested, distilled water and DMSO 1% were used as controls. The larvae were transferred by using a 20 μL micropipette with a drop of water in a 24-well polystyrene clear flat bottom plate with a lid, provided with 50 μL of 5% insect diet and exposed to a total volume of 2 mL of compound solutions and controls for each well. The number of living larvae was recorded 24 and 48 h post treatment. The larvae that showed no signs of movement after probing with a needle were considered dead. Bioassays were conducted at 27 ± 1 °C, 90 ± 5% relative humidity (R.H.) and a photoperiod of 14:10 L:D.

Based on the results of this initial screening, new bioassays were conducted to evaluate the effects of cyclopaldic acid, 1,2-*O*,*O*’-diacetyllycorine, *N*-methyllycorine iodide and α-dihydrolycorine, tested at increasing dosages on the development of *Ae*. *albopictus* until adult emergence. All compounds, except 1,2-*O*,*O*’-diacetyllycorine, were tested at 6.125, 12.5, 25, 50 and 100 ppm, for solubility problems 1,2-*O*,*O*’-diacetyllycorine was not tested at 100 ppm. The insecticide Device^®^ SC-15 (based on Diflubenzuron) for mosquito larvae was used as a positive control and was tested at 7, 12.5, 25, 50 and 100 ppm. Twenty first-instar larvae were transferred in 100-mL beakers, provided with 100 μL of 5% insect diet and were exposed to compound solutions and to controls. Five replicates were utilized for each concentration, for Device^®^ SC-15, as well as for the controls. The number of living insects was recorded every 24 h from the first-instar to adult emergence. The larval mortality percentages, obtained at 24 and 48 h, were reported as an average of values from five replicates, corrected using Abbotts’s formula [73]. For calculating LC_50_ and LC_90_ at 95% confidence interval, the data obtained by the larval mortality at 24 and 48 h were corrected using Abbott’s formula, transformed into arcsine/proportion values and then were subjected to probit regression analysis [74,75]. In addition, for calculating LC_50_ and LC_90_ at 95% confidence interval, the data obtained by the larval mortality at 24 and 48 h were subjected to probit regression analysis without any kind of transformation and without correction for mortality.

The total larval and pupal mortality was estimated by counting the dead samples during the entire bioassay. Larval mortality was expressed in percentage according to the initial number of larvae, pupal mortality percentage was estimated according to the total number of pupae obtained. The total number of days from the start of the bioassay, on which dead larvae were recorded, and the total number of days from the pupation, on which dead pupae were recorded, were also reported. The mean larval duration was obtained by multiplying the number of days, exerted by the larvae to develop in pupae, by the number of pupae obtained on these days in each replicate; these values were summed and the total was divided by the total number of larvae developed in pupae. The mean pupal duration was obtained by multiplying the number of days, exerted by the pupae to develop in adults, by the number of adults obtained in each replicate; these values were summed and the total was divided by the total number of pupae developed in adults.

The mean larval development time values obtained in the control bioassays with distilled water and DMSO 1% were analyzed by Student’s *t*-test (*p* = 0.05) for independent samples. The same statistical analysis was carried out on distilled water and DMSO 1% pupal duration values. The non-parametric Kruskal-Wallis test for multiple independent comparisons followed by pairwise Mann–Whitney U-test comparisons (*p* < 0.05) were used to compare the larval and the pupal duration values obtained in the bioassays with DMSO 1% and with each of the compounds tested.

2-Methyl-1,4-naphtoquinone, 2-hydroxy-1,4-naphtoquinone and 2-methoxy-1,4-naphtoquinone were tested at 100, 50, 25, 12.5 and 6.125 ppm towards third-instar larvae. Distilled water and DMSO 1% were used as controls and the insecticide Device^®^ SC-15 as the positive control. The larvae were transferred in 100-mL beakers, were exposed to test compounds and the number of dead larvae in each beaker was recorded 24, 48 and 72 h after the start of the bioassays. Five replicates, each consisting of 20 three-instar larvae, were utilized for each concentration as well as for the controls. In naphthoquinones tests, no mortality was detected in controls after exposure, so no correction was required based on Abbott’s formula. The mean of the mortality percentages, at each concentration, was determined. The values of dead larvae obtained by the bioassays, at different concentrations, were subjected to probit regression analysis for estimation the mean lethal concentration values (LC_50_ and LC_90_) at 95% confidence interval [74].

The raw data on larval-pupal survival obtained after 24, 48 and 72h after the start of the 1,4-naphthoquinone structural derivatives and Device^®^ SC-15 bioassays were analyzed using the General Linear Model (GLM) for repeated measures (over time) procedure and compared by using a one-way correlated analysis of variance (Tests of within-subjects effects). The differences between the means of the number of survivors in each of the bioassays, carried out using different concentrations of 1,4-naphthoquinone structural analogues and of Device^®^ SC-15, and the means of the number of survived larvae-pupae of related controls over time were analyzed and adjusted with Bonferroni test [76] for multiple comparisons. The Bonferroni test was also used to assess whether the mean number of larvae and pupae surviving to exposure to the same concentration of 1,4-naphthoquinone structural derivatives and of Device^®^ SC-15 and the mean number of larvae and pupae surviving in control solution, at different time of exposure, were significantly different.

All the statistical analyses were performed by Statistical Package for Social Sciences (SPSS), version 20.0 for Windows software (SPSS Inc., Chicago, IL, USA).

## Figures and Tables

**Table 1:** Effect of cyclopaldic acid, *N*-methyllycorine iodide, Device^®^SC-15 against *Ae*. *albopictus* first-instar larvae. ^1^

LC_50_ (ppm) (LCL-HCL) 24 h	LC_90_ (ppm) (LCL-HCL) 24 h	χ^2^ (df)	LC_50_ (ppm) (LCL-HCL) 48 h	LC_90_ (ppm) (LCL-HCL) 48 h	χ^2^ (df)
**Cyclopaldic acid**					
113.8	256.0	11.4 (28)	40.1	105.1	30.6 (28)
(91.3–157.7)	(198.5–374.6)		(34.6–46.3)	(92.7–122.4)	
102.6 *	202.5 *	12.9 (28) *	28.1 *	60.3 *	99.0 (28) *
(87.5–126.3)	(168.6–258.7)		(22.1–35.6)	(49.7–79.4)	
***N*-Methyllycorine iodide**					
177.6	380.8	11.5 (23)	78.5	176.2	23.1 (23)
(126.3–357.6)	(257.4–827.1)		(67.4–94.8)	(147.4–224.4)	
176.6 *	332.5 *	7.3 (23) *	64.5 *	128.8 *	30.5 (23) *
(132.7–294.1)	(240.0–587.1)		(56.3–74.9)	(111.7–154.9)	
**Device^®^ SC-15**					
18.9	104.8	4.3 ^a^ (23)			
(8.0–26.3)	(88.2–132.1)				
8.8 *	59.3 *	12.2 ^a^ (23) *			
(1.2–14.3)	(50.8–72.6)				

^1^ Probit regression analysis (LC_50_ and LC_90_) at 95% confidence interval, at 24 and 48 h post treatment, obtained in the bioassay to evaluate the effects of cyclopaldic acid, *N*-methyllycorine iodide and Device^®^ SC-15 on *Ae*. *albopictus* first-instar larvae; LC_50_ = lethal concentration (ppm) that kills 50% of the exposed larvae; LC_90_ = lethal concentration (ppm) that kills 90% of the exposed larvae; ^a^ = Since goodness-of-fit test is significant (*p* < 0.05), a heterogeneity factor is used in the calculation of confidence limits (CL). * Data with asterisk were obtained without correction for mortality.

**Table 2:** Effects of cyclopaldic acid, 1,2-*O*,*O*’-diacetyllycorine, *N*-methyllycorine iodide, and α-dihydrolycorine on the development of *Ae*. *albopictus*. ^1^

Compound Concentration (ppm)	Larval Mortality (%) ^2^	Mean Larval Duration (Days) ± SE	Pupal Mortality (%) ^3^	Mean Pupal Duration (Days) ± SE
**Control (distilled water)**				
	7.0 (7)	6.4 ± 3.5 a	2.1 (4)	3.5 ± 2.3 a
**Control (DMSO 1%)**				
	7.0 (7)	6.6 ± 3.0 a	4.3 (4)	3.8 ± 1.9 a
**Cyclopaldic acid**				
100	100.0 (3)			
50	100.0 (5)			
25	79.0 (11)	8.6 ± 1.4 b	38.1 (5)	3.1 ± 2.5 b
12.5	38.0 (28)	8.5 ± 2.5 b	16.1 (5)	3.6 ± 1.0 b
6.25	21.0 (16)	7.6 ± 2.5 b	15.2 (9)	3.6 ± 1.2 ab
3.125	13.0 (8)	7.3 ± 2.6 b	11.5 (7)	3.0 ± 1.2 ab
**1,2-*O*,*O*’-Diacetyllycorine**				
50	98.0 (14)			
25	62.0 (7)	7.3 ± 2.0 b	7.9 (7)	3.3 ± 0.9 b
12.5	44.0 (12)	7.1 ± 2.3 b	7.1 (5)	2.9 ± 0.8 b
6.25	34.0 (7)	7.1 ± 2.5 b	12.3 (7)	4.1 ± 1.3 b
***N*** **-methyllycorine iodide**				
100	100.00 (10)			
50	75.0 (14)	7.2 ± 1.4 b	48.0 (6)	4.5 ± 1.1 cb
25	25.0 (25)	7.4 ± 2.0 b	12.0 (10)	4.8 ± 1.2 a
12.5	21.0 (22)	7.3 ± 1.7 b	12.7 (7)	3.9 ± 1.3 ab
6.25	16.0 (17)	7.2 ± 1.8 a	13.1 (6)	4.4 ± 1.2 ab
***α*** **-Dihydrolycorine**				
100	100.0 (9)		0.00	
50	100.0 (9)		0.00	
25	12.0 (15)	6.9 ± 3.1 a	5.7 (7)	4.3 ± 1.7 a
12.5	13.0 (7)	7.2 ± 3.6 a	5.7 (5)	4.1 ± 1.1 a
6.25	10.0 (5)	6.9 ± 2.7 a	4.4 (3)	3.9 ± 1.3 a
**Device^®^ SC-15**				
100	100.0 (1)			
50	100.0 (2)			
25	100.0 (2)			
12.5	100.0 (2)			
7	100.0 (2)			

^1^ Larval and pupal mortality obtained in bioassays with distilled water, DMSO 1%, cyclopaldic acid, 1,2-*O*,*O*’-diacetyllycorine, *N*-methyllycorine iodide, α-dihydrolycorine, and Device^®^ SC-15, tested at different concentrations, on the development of *Ae*. *albopictus*. The larval and pupal duration values obtained in the bioassays with DMSO 1% and with each of the compounds tested were analysed by a non-parametric Kruskal–Wallis test for multiple independent comparisons, with subsequent pair-wise Mann-Whitney U-test comparisons (*p* < 0.05). Different letters indicate significant differences (*p* < 0.05); ^2^ The number of days, from the start of the bioassay, on which dead larvae were recorded; ^3^ The number of days, from the pupation, on which dead pupae were recorded.

**Table 3:** Larvicidal activity of 2-methyl-1,4-naphthoquinone, 2-hydroxy-1,4-naphthoquinone and Device^®^ SC-15 against *Ae*. *albopictus* third-instar larvae. ^1^

LC_50_ (ppm) (LCL-HCL) 24 h	LC_90_ (ppm) (LCL-HCL) 24 h	χ^2^ (df)	LC_50_ (ppm) (LCL-HCL) 48 h	LC_90_ (ppm) (LCL-HCL) 48 h	χ^2^ (df)	LC_50_ (ppm) (LCL-HCL) 72 h	LC_90_ (ppm) (LCL-HCL) 72 h	χ^2^ (df)
**2-Methyl-1,4-naphthoquinone**								
24.2	39.4	35.8 ^a^ (23)	14.5	20.7	11.6 (23)	12.1	15.8	10.2 (23)
(21.6–27.1)	(35.3–45.4)		(13.5–15.6)	(19.1–22.9)		(11.5–12.9)	(14.7–17.7)	
**2-Hydroxy-1,4-naphthoquinone**								
33.7	41.0	1.8 (23)	31.9	39.2	0.8 (23)	30.9	39.6	13.9 (23)
(31.2–38.0)	(37.0–48.5)		(29.3–38.4)	(34.5–52.0)		(28.8–34.1)	(35.9–46.1)	
**Device^®^ SC-15**								
50.6	95.3	60.2 ^a^ (23)	36.4	90.6	43.4 ^a^ (23)	15.0	58.9	26.2 (23)
(42.7–60.1)	(81.9–116.5)		(28.8–44.4)	(77.0–112.2)		(9.7–19.5)	(51.3–70.2)	

^1^ Probit regression analysis (LC_50_ and LC_90_) at 95% confidence interval, obtained in larvicidal test conducted with 2-methyl-1,4-naphthoquinone, 2-hydroxy-1,4-naphthoquinone and Device^®^ SC-15 against *Ae*. *albopictus* third-instar larvae; LC_50_ = lethal concentration (ppm) that kills 50% of the exposed larvae; LC_90_ = lethal concentration (ppm) that kills 90% of the exposed larvae. ^a^ = Since goodness-of-fit test is significant (*p* < 0.05), a heterogeneity factor is used in the calculation of confidence limits (CL).

**Table 4:** Effect of 2-methyl-1,4-naphthoquinone, 2-hydroxy-1,4-naphthoquinone and 2-methoxy-1,4-naphthoquinone and Device^®^ SC-15, at different concentrations, on survival of *Ae. albopictus* third-instar larvae and pupae. ^1^

CompoundConcentration (ppm)	GLM (Time × Treatment) ^a^		Bonferroni Test ^b^ Mean Treatment	Mean Control	Statistical Significance
**2-Methyl-1,4-naphthoquinone**					
**6.25**	*F*_2.16_ = 2.7	*p* > 0.05	19.6 ± 0.2	20.0 ± 0.2	n.s.
**12.5**	*F*_2.16_ = 55.6	*p* < 0.01	12.5 ± 0.1	20.0 ± 0.1	**
**25**	*F*_2.16_ = 297.8	*p* < 0.01	2.2 ± 0.1	20.0 ± 0.1	**
**50**	*F*_2.16_ = 36.0	*p* < 0.01	0.4 ± 0.05	20.0 ± 0.05	**
**100**			0.0 ± 0.0	20.0 ± 0.0	**
**2-Hydroxy-1,4-naphthoquinone**					
**6.25**	*F*_2.16_ = 1.0	*p* > 0.05	19.9 ± 0.05	20.0 ± 0.05	n.s.
**12.5**	*F*_2.16_ = 2.7	*p* > 0.05	19.9 ± 0.06	20.0 ± 0.06	n.s.
**25**	*F*_2.16_ = 33.8	*p* < 0.01	17.5 ± 0.2	20.0 ± 0.2	**
**50**			0.0 ± 0.0	20.0 ± 0.0	**
**100**			0.0 ± 0.0	20.0 ± 0.0	**
**2-Methoxy-1,4-naphthoquinone**					
**6.25**	-	-	19.8 ± 0.1	20.0 ± 0.1	n.s.
**12.5**	*F*_2.16_ = 1.0	*p* > 0.05	19.9 ± 0.09	20.0 ± 0.09	n.s.
**25**	*F*_2.16_ = 1.0	*p* > 0.05	19.7 ± 0.2	20.0 ± 0.2	n.s.
**50**	*F*_2.16_ = 28.2	*p* < 0.01	16.7 ± 0.6	20.0 ± 0.6	**
**100**			0.0 ± 0.00	20.0 ± 0.0	**
**Device^®^ SC-15**					
**7**	*F*_2.16_ = 351.4	*p* < 0.01	16.0 ± 0.3	20.0 ± 0.3	**
**12.5**	*F*_2.16_ = 77.5	*p* < 0.01	14.1 ± 0.3	20.0 ± 0.3	**
**25**	*F*_2.16_ = 51.1	*p* < 0.01	12.2 ± 0.3	20.0 ± 0.3	**
**50**	*F*_2.16_ = 10.7	*p* < 0.01	3.5 ± 0.5	20.0 ± 0.5	**
**100**	*F*_2.16_ = 12.3	*p* < 0.01	2.1 ± 0.3	20.0 ± 0.3	**

^1^ GLM values describe the effect of time on survival of the larvae-pupae; ns = not significant; * *p* < 0.05; ** *p* < 0.01; ^a^ Values of *p* > 0.05 for GLM indicate that the interaction between the two conditions (treated and control) and the change over time were not statistically significant. Values of *p* < 0.05 for GLM indicate that the interaction between the two conditions (treated and control) and the change over time were statistically significant; ^b^ Differences between the means of the number of survived larvae-pupae at different 1,4-naphtoquinone structural derivatives and concentrations in each of the experimental treatments and those of the number of related controls over time were analyzed and adjusted with Bonferroni test for the multiple of comparisons.

## Data Availability

Data is contained within the article.

## Supplementary Materials

The following are available online at https://www.mdpi.com/article/10.3390/toxins13040285/s1, Table S1: Larvicidal activity of α-dihydrolycorine against *Ae*. *albopictus* first-instar larvae., Table S2: Larvicidal activity of 2-methoxy-1,4-naphthoquinone against *Ae*. *albopictus* third-instar larvae.
