# Supplementary material for: Activity of Some Plant and Fungal Metabolites towards Aedes albopictus (Diptera, Culicidae)
Source: Toxins (Basel). 2021 Apr 18;13(4):285. doi: 10.3390/toxins13040285 (PMC8073068; doi:10.3390/toxins13040285)
Supplement: Supplementary file 1 [file toxins-13-00285-s001.zip › toxins-1114629-supplementary.pdf]

Supplementary Materials: Activity of Some Plant and Fungal Metabolites Towards *Aedes albopictus* (Diptera, Culicidae)

Sonia Ganassi, Marco Masi, Pasqualina Grazioso, Antonio Evidente and Antonio De Cristofaro

Table S1. Larvicidal activity of α-dihydrolycorine against *Ae. albopictus* first-instar larvae.

**Concentration (ppm)**	**Mean mortality (%) ± SE 24 h**
100	47.4 ± 1.6
50	46.5 ± 1.9
25	8.05 ± 1.9
12.5	2.0 ± 1.2
6.25	1.0 ± 1.0
	48 h
100	46.8 ± 2.2
50	57.0 ± 4.6
25	9.0 ± 2.4
12.5	3.0 ± 1.2
6.25	2.0 ± 1.2

Mean mortality percentages at 24 and 48 h post treatment, obtained in the bioassay to evaluate the effects of α-dihydrolycorine on *Ae. albopictus* first-instar larvae.

Table S2. Larvicidal activity of 2-methoxy-1,4-naphthoquinone against *Ae. albopictus* third-instar larvae.

**Concentration (ppm)**	**Mean mortality (%) ± SE 24 h**
100	100 ± 0.0
50	5.0 ± 2.6
25	1.0 ± 1.0
12.5	0.0 ± 0.0
6.25	1.0 ± 1.0
	Mean mortality (%) ± SE 48 h
100	100 ± 0.0
50	9.0 ± 3.1
25	1.0 ± 1.0
12.5	1.0 ± 1.0
6.25	1.0 ± 1.0
	Mean mortality (%) ± SE 72 h
100	100 ± 0.0
50	30.0 ± 4.9
25	2.0 ± 1.4
12.5	1.0 ± 1.0
6.25	1.0 ± 1.0

Mean mortality percentages at 24, 48 and 72 h post treatment, obtained in the bioassay to evaluate the effects of 2-methoxy-1,4-naphthoquinone on *Ae. albopictus* third-instar larvae.
